# Microbial Community Structure in Lake and Wetland Sediments from a High Arctic Polar Desert Revealed by Targeted Transcriptomics

**DOI:** 10.1371/journal.pone.0089531

**Published:** 2014-03-03

**Authors:** Magdalena K. Stoeva, Stéphane Aris-Brosou, John Chételat, Holger Hintelmann, Philip Pelletier, Alexandre J. Poulain

**Affiliations:** 1 Department of Biology, University of Ottawa, Ottawa, Ontario, Canada; 2 Department of Mathematics and Statistics, University of Ottawa, Ottawa, Ontario, Canada; 3 Environment Canada, National Wildlife Research Centre, Ottawa, Ontario, Canada; 4 Department of Chemistry, Trent University, Peterborough, Ontario, Canada; U. S. Salinity Lab, United States of America

## Abstract

While microbial communities play a key role in the geochemical cycling of nutrients and contaminants in anaerobic freshwater sediments, their structure and activity in polar desert ecosystems are still poorly understood, both across heterogeneous freshwater environments such as lakes and wetlands, and across sediment depths. To address this question, we performed targeted environmental transcriptomics analyses and characterized microbial diversity across three depths from sediment cores collected in a lake and a wetland, located on Cornwallis Island, NU, Canada. Microbial communities were characterized based on 16S rRNA and two functional gene transcripts: *mcrA*, involved in archaeal methane cycling and *glnA*, a bacterial housekeeping gene implicated in nitrogen metabolism. We show that methane cycling and overall bacterial metabolic activity are the highest at the surface of lake sediments but deeper within wetland sediments. Bacterial communities are highly diverse and structured as a function of both environment and depth, being more diverse in the wetland and near the surface. Archaea are mostly methanogens, structured by environment and more diverse in the wetland. *McrA* transcript analyses show that active methane cycling in the lake and wetland corresponds to distinct communities with a higher potential for methane cycling in the wetland. *Methanosarcina* spp., *Methanosaeta* spp. and a group of uncultured Archaea are the dominant methanogens in the wetland while *Methanoregula* spp. predominate in the lake.

## Introduction

In response to climate warming, northern aquatic ecosystems are rapidly changing. This change begins as an alteration of the landscape (e.g., retrogressive thaw slumps), which in turn affects the hydrology as well as the cycling of organic carbon, contaminants and other nutrients [1]–[3]. Microbial communities play a dominant role in the geochemical cycling of nutrients (e.g., carbon) and contaminants (e.g., mercury) in anaerobic freshwater sediments [4], [5]. For instance, studies have characterized microbial processes involved in carbon cycling and methane production and have identified their drivers in various Arctic locations, such as terrestrial (*e.g.*, [6]–[8]), aquatic (*e.g.*, [9]) and more recently, subglacial environments (*e.g.*, [10]). Methanogens are abundant in cold environments [11] and *Methanosaetaceae*, *Methanosarcinaceae*, *Methanobacteriaceae* and *Methanomicrobiales* are often identified as the dominant methane producers in Arctic wetlands [12]. Furthermore, investigations on mercury methylation in high Arctic wetlands revealed that these environments are sources of monomethylmercury (MMHg) to downstream aquatic ecosystems [13]. Recent data underscore the diversity of potential mercury methylators including not only the well-known sulfate- (SRB) and iron- (FeRB) reducing bacteria, but also methanogenic archaea, syntrophic, acetogenic, and fermentative *Firmicutes* [14]. These recent findings highlight the necessity to carefully assess the presence and activity of these microbial communities when quantifying the flux of such contaminants through the environment [15]–[17]. However, little is known about the structure and identity of active members of bacterial and archaeal communities from aquatic ecosystems set in high Arctic polar deserts. Additionally, the question of how microbial communities are vertically structured within sediments in these environments has also received little attention. This is a critical gap in our basic knowledge of microbial processes at high latitudes: polar deserts are particularly sensitive to climate change [18], [19], and aquatic ecosystems have already started to be irreversibly altered [20] and are increasingly subject to deposition of anthropogenic contaminants, such as Hg [3].

To address this knowledge gap, we performed targeted environmental transcriptomics analyses across depth from sediment cores collected in a lake and a wetland, both located in the high Arctic on Cornwallis Island, NU, Canada. Char Lake is a small, oligotrophic lake located near the hamlet of Resolute Bay, NU, Canada that has been extensively studied over the last 40 years [21] and has recently started to show signs of change consistent with recent climatic changes [22]. Patchy wetlands are a common feature of polar desert landscapes and typically maintain a relatively lush vegetation cover that stands in stark contrast to the surrounding barren surfaces [23]. Our survey of molecular diversity of microbes thriving in polar desert lake and wetland sediments highlights a very complex community structure with previously uncharacterized microbial players inferred to exhibit diverse methane cycling strategies. Our novel approach, based on RNA amplification from freshwater sediments, highlights the importance of combining targeted functional gene transcript analyses with environmental genomics in order to gain a functional perspective on the composition of microbial communities, particularly when studying transient environments in the high Arctic.

## Materials and Methods

### Sampling Locations and Procedures

Sediment cores were sampled in August 2010 from two locations on Cornwallis Island, Nunavut, Canada: Char Lake (74°45′45.30 N–94°53′50.53 W) and Small Lake Wetland (74°45′45.30 N–95°04′39.07 W). Sampling occurred on federal Crown land and sites on Cornwallis Island (NU) were accessed with the authorization of the Nunavut Research Institute under research license 02-109-11R as well as with consent from the Resolute Bay Hunter's and Trapper's Association. This study was part of a bigger project that aimed at determining methylmercury cycling in high Arctic lake sediments. The field studies did not involve endangered or protected species. We collected two cores in the wetland and three cores in the lake. Char Lake is a small (area = 52.6 ha, mean depth = 10.2 m, maximum depth = 27.5 m) monomictic polar lake (constantly below 4°C) located near the hamlet of Resolute Bay. The lake mixes once a year during a brief summer turnover period, but during winter, surface sediments become anoxic [24]. Continuous permafrost underlies the study site and ground thaw can reach about 100 cm but in most wetland sites, active layer depths are often limited to 50 cm. Thickness of organic soil layer ranges from 5 to 12.5 cm [25]. The drainage basin is sparsely vegetated, with plant cover representing only 5–7% of the total area [21] (Photograph S1 in File S1). Sediment cores were collected using a gravity corer and a 7.62 cm diameter by 60 cm long polycarbonate core tube beveled at one end. The polycarbonate core tube was pre-sliced every 1 cm and rings were re-assembled together using tape on the outside. The main advantage of this design is that sediment compaction typically observed using traditional extruding technique, was avoided. After collection, sediment cores were kept in the dark at 4°C for no longer than 12 h until sectioning corresponding to the incubation time required to the assessment of methylmercury cycling. The core was sectioned every centimeter using ethanol washed stainless steel razor blades and an acid and ethanol washed thin sheet of PFA teflon. Immediately after sectioning, sediments from 1 cm, 3 cm and 10 cm depths were added to a sterile 5 mL cryovial and placed in a dry shipper pre-incubated with liquid nitrogen and holding a temperature ca. −150°C. Sediments were brought back to the lab under cryogenic conditions and transferred to a −80°C freezer until processed for DNA and RNA extraction.

Patchy wetlands are commonly found in polar deserts. Small Lake Wetland is a small pond that likely shrunk and accumulated microbial mats and vascular plants (Photograph S2 in File S1). Such wetlands are fed by late lying snow banks and can dry up by the end of the summer [23], [26]. The wetland was sampled following the same protocol as the lake. Basic water chemistry data for the lake and the wetland are provided in Table S1 in File S1 as well as pictures of the sampling sites as supporting information.

### Extraction of DNA and RNA

Samples from the depths of interest were immediately frozen at −150°C and held in a dry shipper after sectioning. However, due to limitation associated with the logistics of fieldwork in the high Arctic, we were limited by the amount of sedimentary material that could be preserved under cryogenic conditions. For this reason, while quantitative DNA analyses were performed on replicate cores, RNA analyses were only performed on one core at each sampling site. We focused on RNA, because DNA can either be extracted from non-viable organisms [27] or exist as free molecules adsorbed onto sediment particles [28]. Upon thawing, 5 g of sediment samples were initially washed using 5 mL of 10 µM EDTA pH = 8.0, 50 µM TrisHCl pH = 8.0 and 50 µM Na_2_HPO_4_ pH = 8.0 (washing buffer adapted from [29]). This washing step was required to limit the amount of potential PCR inhibitors carried over in downstream steps. All solutions were prepared in diethylpyrocarbonate (DEPC) treated water to inactivate RNase enzymes. Samples amended with the wash buffer were vortexed for 30 sec and spun at 10,000 g for 5 min. The washing step was repeated 3 times. DNA was extracted from each of the 3 depths of interest for 3 cores for the lake and 2 cores for the wetland, using a Mobio Power Soil DNA Isolation Kit following the manufacturer's instructions. From each of the 3 depths, total RNA was extracted using the RNA Powersoil Total RNA Isolation Kit (MoBio, Cat#12866), from the wetland and the lake cores. RNA was treated with RQI RNase-Free DNase (Promega Cat#M610A) followed by RNeasy MiniElute Cleanup (Qiagen Cat#74204).

Reverse transcription was carried out on total RNA using SuperScript III (Invitrogen Cat#18080-051) and random hexamers, following the manufacturer's instructions.

### Amplification of RNA and cDNA synthesis

Although both 16S rRNA and *glnA* gene transcripts were detected as discrete bands on an agarose gel when testing for their presence in the pool of cDNA, our initial attempts at detecting *mcrA* transcripts were unsuccessful. This may be associated with the very low organic content of Char Lake surface sediments, indicative of low microbial biomass. Because we could not obtain a visible discrete band on an agarose gel when testing for the presence of *mcrA* transcripts in the pool of cDNA, we decided to use RNA amplification of environmental samples [30]. This was achieved using *in-vitro* transcription mediated linear amplification of RNA with the MessageAmp II-Bacteria Kit (Ambion Cat#AM1790) and a 14 h amplification time. The details of the procedure for RNA analyses are presented in Figure S1 in File S1.

Reverse transcription was carried out on amplified RNA using SuperScript III (Invitrogen Cat#18080-051) and random hexamers, following the manufacturer's instructions. Starting in all cases from 1 µg of amplified RNA, cDNA concentrations for each sample, measured by absorption on a Nanodrop 2000 were: 497.2 ng/µl, 505.7 ng/µl and 511.1 ng/µl for the lake at 1, 3 and 10 cm, respectively and 507.7 ng/µl, 499.2 ng/µl and 508.5 ng/µl for the wetland at 1, 3 and 10 cm, respectively. cDNA synthesis efficiency showed very little variation among samples with cDNA concentrations for each site varying less than 3% for the lake and less than 2% for the wetland. Subsequent quantitative and diversity analyses for all the transcripts tested and presented in this study were performed on cDNA synthesized from the amplified RNA.

### Conventional and quantitative PCR

We performed conventional and quantitative PCR (qPCR) on DNA and RT-qPCR on cDNA targeting 16S rRNA (Bacterial 16S rRNA: 880 bp, forward primer 27F: 5′-AGA GTT TGA TCM TGG CTC AG-3′ and reverse primer 907R: 5′-CCG TCA ATT CMT TTR AGT TT-3′; Archaeal 16S rRNA, forward primer 109F: 5′-ACK GCT CAG TAA CAC GT-3′, and reverse primer 915R: 5′-GTG CTC CCC CGC CAA TTC CT-3′), *glnA* (154 bp, forward primer glnA-F: 5′-GAT GCC GCC GAT GTA GTA-3′, reverse primer glnA-R: 5′-AAG ACC GCG ACC TTY ATG CC-3′ [31]) and *mcrA* (750 bp, ME1 forward primer: 5′-GCM ATG CAR ATH GGW ATG TC-3′, ME2 reverse primer: 5′-TCA TKG CRT AGT TDG GRT AGT-3′ [32]) genes and transcripts. The *glnA* gene encodes the glutamine synthetase and is used here as a marker for core housekeeping bacterial metabolic activity [33]. The *mcrA* gene encodes the alpha subunit of the methylcoenzyme-M reductase enzyme. While *mcrA* sequence data have been used as a proxy for methane production (methanogenesis) [34], [35], the methylcoenzyme-M reductase enzyme is also expected to be involved in methane destruction via anaerobic methane oxidation [36], [37].

Conventional PCRs were run using GoTaq from Promega and the following cycling conditions: *glnA*: 94°C for 2 min , (94°C for 30 sec, 60°C for 30 sec, 72°C for 20 sec)×30 cycles, 72°C for 5 min; 16S rRNA: 94°C for 2 min, (94°C for 30 sec, 48°C for 30 sec, 72°C for 1 min)×30 cycles, 72°C for 5 min; *mcrA*: 94°C for 2 min, (94°C for 30 sec, 48.8°C for 30 sec, 72°C for 1 min)×30 cycles, 72°C for 5 min.

Partial gene sequences were amplified from genomic DNA using primers described above to construct calibration curves for absolute gene quantification. For this purpose, DNA was extracted from *Methanosarcina acetivorans* to amplify *mcrA* and archaeal 16S rRNA genes and from *Pseudomonas aeruginosa* to amplify *glnA* and bacterial 16S rRNA genes. Amplicons were gel purified with a Qiagen QiaexII Purification Kit (Qiagen Cat#20021) and cloned using the StrataClone PCR Cloning Kit (StrataClone PCR Clonning Kit Cat#240205), as per the manufacturer's instructions. Plasmids were purified using the Promega Wizard Plus SV Miniprep Kit (Promega Cat#A1330) and quantified using a Thermo Nanodrop 2000. All dilutions and all samples were run in triplicates. We used a recombinant DNA calibration curve to assess transcript copy number [38]. With this calibration curve model, only the existing cDNA molecules derived from reverse transcription can be quantified and not the existing mRNA molecules present in the native total RNA samples. We used this approach because (i) we performed RNA amplification and (ii) the cDNA synthesis efficiency was very similar for all samples (<3% variability for the lake and 2% for the wetland). While this approach does not provide absolute transcript copy number in the native pool of mRNA, it is appropriate for gene-specific relative abundance comparison.

**Figure 1:**
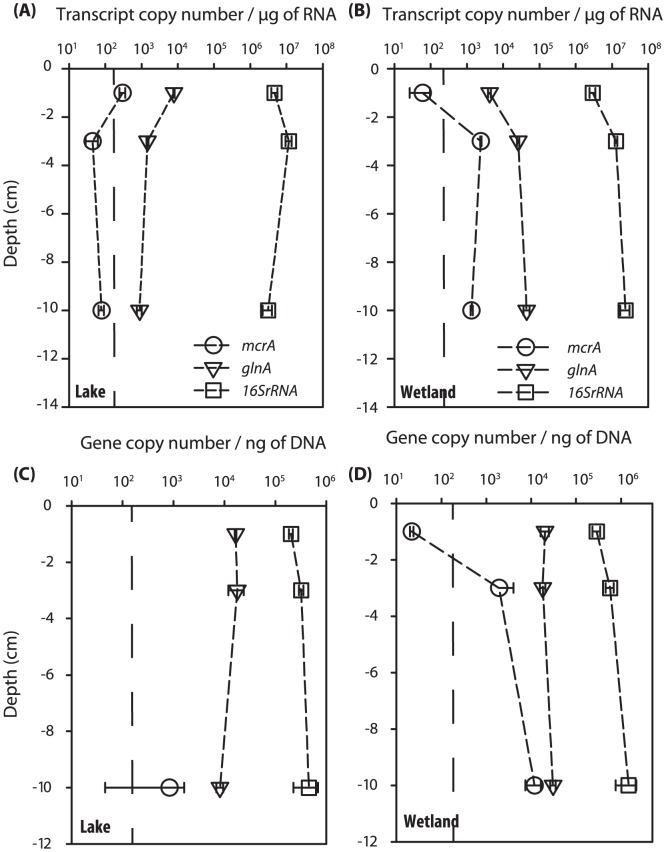
Depth profiles showing copy numbers in the pool of cDNA for *mcrA*, *glnA* and bacterial 16S rRNA in lake (A) and wetland (B) environments, and gene copy numbers for *mcrA*, *glnA* and 16S rDNA in the lake (C) and wetland (D). Vertical dashed line indicates the limit of quantification for the assay for *mcrA* gene transcripts. Error bars represent 1 SD.

The SsoFast EvaGreen Supermix Kit for real-time PCR was used for all gene and transcript quantification (Bio-Rad Cat#172-5200); reaction conditions were as indicated by the manufacturer. All assays were run on an Eco Illumina real-time PCR system. Primer efficiencies were determined using gene standards as templates. Bacterial 16S rRNA primer efficiency was 97.5% with an *r* ^2^ = 0.99; *glnA* primer efficiency was 97.2% with *r* ^2^ = 0.97; *mcrA* primer efficiency was 86.0% with *r* ^2^ = 0.99. cDNA samples were pooled together to test for primer efficiency on the environmental samples followed by 10 fold serial dilutions. Bacterial 16S rRNA primer efficiency was 94.7% with *r* ^2^ = 0.99; *glnA* primer efficiency was 132.7% with *r* ^2^ = 0.97; *mcrA* primer efficiency was 137.6% with *r* ^2^ = 0.94. The limit of detection for each assay was calculated as the mean plus three times the standard deviation of the measurement from negative controls ([39] 43 copies for *mcrA*, 24 copies for *glnA* and 33.4 copies for 16S rRNA). While the limit of detection quantifies the ability of the qPCR to detect the transcript, we have set our limit of quantification at 10 times the limit of detection (Figure 1, vertical dashed line).

### Clone Library Construction

PCR products used in clone library construction were generated using cDNA originating from amplified RNA, with primers targeting 16S rRNA for Bacteria and Archaea as well as *mcrA* for Archaea (Table S2 in File S1). Partial gene sequences were amplified from environmental cDNA under conditions described above. Amplicons were gel purified with a Qiagen QiaexII Purification Kit (Qiagen cat#20021) and cloned using the StrataClone PCR Cloning Kit (StrataClone PCR Clonning Kit Cat#240205), as per manufacturer's instructions. One cDNA library of 96 clones was constructed per depth (1, 3 and 10 cm) for each site, for both archaeal and bacterial 16S rRNA gene sequences, totaling 12 clone libraries. For each of the 12 bacterial and archaeal 16S clone libraries, 96 clones were randomly selected for sequencing. The *mcrA* clone libraries were generated from only 3 samples (Small Lake Wetland: 3 cm and 10 cm; Char Lake: 1 cm) as no cDNA was detected on a gel after amplification in the other samples. The *mcrA* clones were initially screened using RFLP: each PCR product was digested with HaeIII and EcoRI digestion enzymes and RFLP patterns were compared for an initial estimate of diversity. As a low number of unique patterns were recovered, only 35–42 clones were sent for sequencing per library. All clones were sequenced by Beckman Coulter Genomics, MA, USA.

### Sequence Analyses

All 16S rRNA sequences were first scanned for vector remnants using the PIPELINE function in the Ribosomal Database Project (RDP); vector sequences were removed from the *mcrA* sequences using the vector annotation tool in Geneious v5.4 [40]. Primers from both 16S rRNA and *mcrA* sequences were removed using Geneious' primer annotation tool. Chimeric sequences were identified using UCHIME [41] in MOTHUR [42]. Sequences identified as chimeric were discarded. The 16S rRNA sequences were aligned with the 16S rRNA core alignment downloaded (September 2012) from the SILVA database using MOTHUR [42], while *mcrA* nucleotide sequences were aligned based on the amino acid sequence using TranslatorX [43]. The model of evolution most appropriate to each of our three datasets (Bacteria 16S rRNA, Archaea 16S rRNA and *mcrA*) was determined using jModelTest v.0.1.1 with the Akaike Information Criterion [44]. Distance matrices were constructed in two steps: (i) building a phylogenetic tree in FastTree [45] under the appropriate model of evolution, and (ii) employing this tree to estimate a distance matrix in PAUP [46]. These matrices were imported into MOTHUR for computing richness (Chao1 and ACE) and diversity (Shannon diversity index, and Simpson diversity index) estimates of samples [42]. Diversity was analyzed both as a function of depth within each environment, and as a function of environment, pooling over depths. Operational taxonomic units (OTUs) were defined based on 97% sequence identity [47]. Good's coverage was also calculated [48]. Rarefaction curves, including 95% confidence intervals were constructed to allow for comparison among samples.

Phylogenies were estimated for the bacterial and archaeal environmental 16S rRNA sequences. In each case, trees were reconstructed by maximum likelihood in PhyML v3.0 [49] with the alignments and substitution models obtained above. Support values were estimated using the aLRT SH-like procedure [50]; support values were compared to results obtained with FastTree; the PhyML and FastTree estimates were compared using the Shimodaira Hasegawa (SH) test [51] as implemented in the phangorn library in R [52].

To identify the taxonomic placement of our environmental sequences, on both the bacterial and the archaeal trees, highly supported clades (support values >99%) were visually identified and representative sequences were compared to the NCBI's non-redundant database using BLASTn, the RDP classifier, as well as the SILVA database [53]. New alignments, model selection and trees were generated as above, including both environmental and known sequences. These trees were compared to the trees obtained without the known sequences, by first pruning the known sequences from the trees using the APE library in R, and then performing SH tests.

The trees built with known members and environmental sequences and those built with only environmental sequences were not significantly different from one another (see Table S3 in File S1). Further, trees constructed in PhyML were not significantly different from those constructed in FastTree. Therefore, for the UniFrac analysis, the PhyML trees containing only environmental sequences were used.

Significant differences among microbial communities were determined using UniFrac [54]. In the UniFrac analyses, jackknife cluster analyses were also performed to allow for a visual representation of patterns among samples. The jackknife fraction *J* (fraction of times the node was recovered among 100 replicates) was calculated for each node; the number of sequences kept was set to the number of sequences in the environment with the least number of sequences. A lineage-specific analysis using UniFrac was also performed to determine lineages of Bacteria contributing significantly to differences between environments and among depths. All nucleotide sequences were deposited in GenBank (JQ792250-JQ793375).

Structural (three-dimensional) and protein motif analyses were performed as follows. Representative amino acid sequences from the lake and from the wetland were modeled using the automated mode in SwissModel [55]; a reference sequence (*Methanospirillum hungatei*) was also modeled. Protein models were compared in SwissPDB Viewer [56]. HHblits was used to identify protein sequences [57].

## Results

### Vertical distribution of microbial activity within sediment cores

We used RT-qPCR to characterize microbial activity at three different depths (1, 3 and 10 cm) in lake and wetland sediments. Two functional genes were targeted: *mcrA* and *glnA*, for which transcript abundances are used as proxies for methane cycling and overall bacterial activity, respectively [33], [35]. Non-coding sequences (Bacterial 16S rRNA) were also quantified. In the lake, *mcrA* transcripts were quantifiable at a depth of 1 cm and although they could be detected at depths of 3–10 cm, they were below our limit of quantification (Figure 1A). In contrast, *mcrA* transcripts in the wetland were least abundant at the surface (detectable but below our limit of quantification) and increased by almost 2 orders of magnitude at depths of 3 and 10 cm (Figure 1B).

The relative abundance of transcripts tested was consistent with what could be expected: *i.e*., *mcrA* < *glnA* << 16S rRNA (Figure 1). Indeed, transcripts of the multiple copy 16S rRNA gene were 3–4 orders of magnitude more abundant than those of the single copy Bacteria *glnA* gene; this is consistent with the fact that Bacteria can harbor up to 16 copies of the 16S rRNA gene per cell [58] and that each cell can contain hundreds of ribosomes [59]. On the other hand, methanogens (as per *mcrA*) were about an order of magnitude less abundant than Bacteria, and both appeared to covary with depth. Critically however, the abundance of methanogens decreased with depth in the lake sediment core (by an order of magnitude), while the opposite was observed in the wetland. These results suggest that methanogenesis and overall bacterial activity are confined to the uppermost layer of lake sediments, while occurring deeper in the wetland.

qPCR data obtained from DNA extracted from replicate cores (Figure 1C and D) were in agreement with what was seen with transcript data; abundances ranked as: *mcrA* < *glnA* << 16S rRNA. Although we noted that in the wetland *mcrA* gene copy number followed the trend observed for the transcripts (*i.e*., below the limit of quantification at the surface, increasing with depth), quantitative gene and transcript data could not be directly compared because of the RNA amplification step performed. This comparison would have allowed for the determination of gene:transcript abundance ratio, reflecting transcript abundance per cell and providing a more direct measure of physiological activity than absolute abundance [35]. Regardless, in the lake, levels of *mcrA* gene and transcripts were generally at or below the detection limit, making quantitative comparisons unreliable.

### Microbial diversity in arctic freshwater sediments

**Figure 2:**
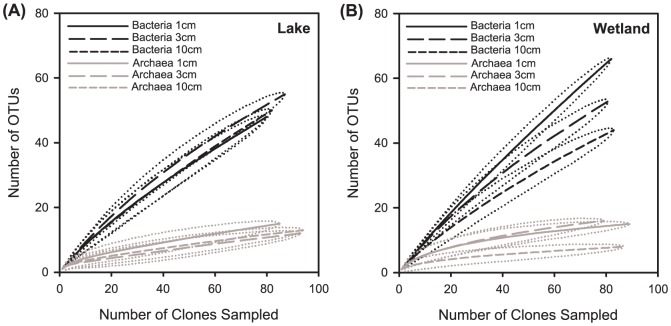
Rarefaction curves for bacterial and archaeal sequences. Results for two environments are shown: (A) lake; (B) wetland. Dotted lines represent the 95% confidence intervals, as computed by resampling in MOTHUR.

Diversity indices (Shannon and 1/Simpson, as assessed using 16S rRNA data) indicated high bacterial diversity in both wetland and lake sediments (Table 1), with diversity and richness estimators (Chao1 and ACE) decreasing with depth (except in the lake for Bacteria; Table 1). Both sites were characterized by incomplete sampling of the bacterial community with low Good's coverage (56–59%) and non-plateauing rarefaction curves (Figure 2). Within bacterial 16S rRNA wetland and lake libraries, a vast majority of clones represented their own OTU at 97% sequence similarity, indicating a high abundance of singletons. Twenty percent of the bacterial community from the lake remained unclassified in NCBI, RDP and SILVA databases, compared to 11.4% for the wetland (Figure S2 in File S1). Richness was also examined both between environments and as a function of depth, by comparing rarefaction curves and their associated 95% confidence intervals (Figure 2). Bacterial richness was comparable between lake and wetland environments and was vertically structured in the wetland (exhibiting non-overlapping confidence intervals), decreasing with depth, but not in the lake.

**Table 1:** Richness, diversity and coverage of bacterial 16S rRNA and Archeal *mcrA* clone libraries.

	*n*	*N*	Chao1	ACE	Shannon	1/Simpson	Coverage
Bacteria wetland	246	145	385.75 (284.1–516.7)	669 (529–860)	4.57 (4.4–4.7)	58.74 (40.9–104.4)	0.56
1 cm	82	66	341.5 (182.5–717.5)	1068 (748–1537)	4.05 (3.9–4.2)	94.89 (50.7–733.1)	0.29
3 cm	81	53	164.42 (98.4–326.7)	249 (175–367)	3.77 (3.6–4.0)	58.91 (38.6–124)	0.51
10 cm	83	44	102.66 (67.6–189.8)	256 (169–405)	3.18 (2.9–3.5)	11.82 (7.5–27.9)	0.6
Bacteria lake	249	137	428.83 (298.6–663.9)	929 (743–1172)	4.47 (4.3–4.6)	57.60 (42.9–87.8)	0.59
1 cm	80	48	188.6 (101.5–417.6)	277 (183–436)	3.48 (3.2–3.7)	23.06 (13.9–67.3)	0.53
3 cm	87	55	129 (87–229)	210 (150–308)	3.81 (3.6–4.0)	58.82 (38.5–125)	0.55
10 cm	82	50	161.42 (95.4–323.7)	779 (574–1064)	3.49 (3.2–3.8)	22.44 (14.0–56.5)	0.51
Archaea wetland	255	27	49.75 (33.3–109.4)	83 (55–137)	2.06 (1.9–2.2)	4.31 (3.6–5.4)	0.95
1 cm	89	16	26.5 (18.0–70.2)	25 (18–55)	2.12 (1.9–2.3)	6.22 (4.9–8.5)	0.92
3 cm	79	16	30 (18.9–83.1)	44 (29–80)	2.08 (1.8–2.3)	5.72 (4.4.–8.1)	0.9
10 cm	87	8	11 (8.4–31.0)	22 (13–50)	0.97 (0.7–1.2)	1.8 (1.5–2.3)	0.95
Archaea lake	272	31	52 (38.0–96.0)	63 (42–126)	1.8 (1.6–2.0)	3.49 (3.0–4.1)	0.93
1 cm	85	15	30 (18.5–79.2)	45 (19–228)	1.65 (1.4–1.9)	3.48 (2.8–4.7)	0.88
3 cm	93	12	30 (16.0–93.3)	57 (15–793)	1.24 (1.0–1.5)	2.60 (2.3–3.0)	0.9
10 cm	94	13	31 (17.0–94.3)	134 (74–255)	1.40 (1.2–1.7)	2.85 (2.4–3.6)	0.9
*mcrA* Wetland	76	10	16 (11.0–48.0)	20 (13–42)	1.65 (1.4–1.9)	4.03 (3.2–5.4)	0.95
*mcrA* Lake	28	7	8 (7.0–15.0)	8 (7–17)	1.58 (1.2–1.9)	4.01 (2.6–8.5)	0.93

Notes — *n* is number of clones in each library, *N* in number of Operational Taxonomic Unit (OTU) is based on 97% nucleotide identity. Richness and diversity are shown as: Chao1 (richness estimate), ACE (abundance-based coverage estimator), Shannon diversity index and the inverse of Simpson diversity index; 95% confidence intervals are indicated in brackets. Coverage is calculated in MOTHUR using methods described by Good (1953).

On the other hand, archaeal and *mcrA* clone libraries represented a much more complete sampling of microbial diversity (Good's coverage: 93–95%; Table 1), with diversity indices and richness estimators greater for the wetland than for the lake (Table 1). Archaeal OTUs were much more populated, with fewer singletons and doubletons. Almost a third (27.8%) of the archaeal community from the lake remained unclassified in NCBI and SILVA databases compared to 6.3% for the wetland (Figure S3 in File S1). Archaeal diversity and richness were significantly lower than that of Bacteria (Table 1 and Figure 2: non-overlapping 95% confidence intervals). Overall, diversity and richness were similar between the two environments, and did not show any evidence of vertical structure except for the deepest sample in the wetland (Table 1, Figure 2). The majority of the Archaea were methanogenic Euryarchaeota (Figure S3 in File S1).

### Microbial community structure differed between lake and wetland sediments

#### Bacteria

**Figure 3:**
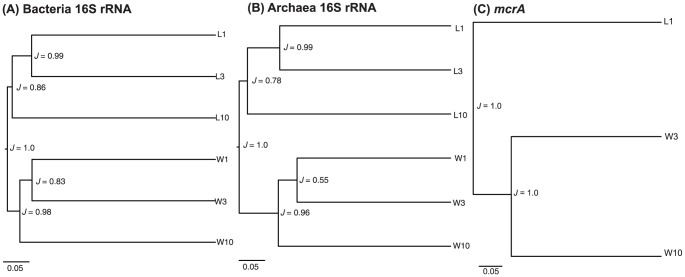
UniFrac clustering of environments based on genetic distance. Three clusters are shown: (A) bacterial 16S; (B) archaeal 16S; (C) *mcrA* sequences. Samples are letter-coded: L for lake and W for wetland environments; numbers indicate sampling depths of 1, 3 and 10 cm. Scale bars show the distance between clusters in UniFrac units. Numbers adjacent to each node indicate the fraction *J* of times this node was recovered among 100 replicates.

Wetland and lake bacterial community structures were significantly different from one another (UniFrac significance analysis: *p*≤0.01). The node separating the lake and wetland bacterial community was recovered 100% of the time with Jackknife resampling (*J* = 1.0), further supporting that both environments are different (Figure 3A). More critically, bacterial communities clustered as a function of depth in the lake (Figure 3A), with community structures at 1 and 3 cm being more similar (*i.e*. sharing more of the total tree branch length) than the one at 10 cm.

**Figure 4:**
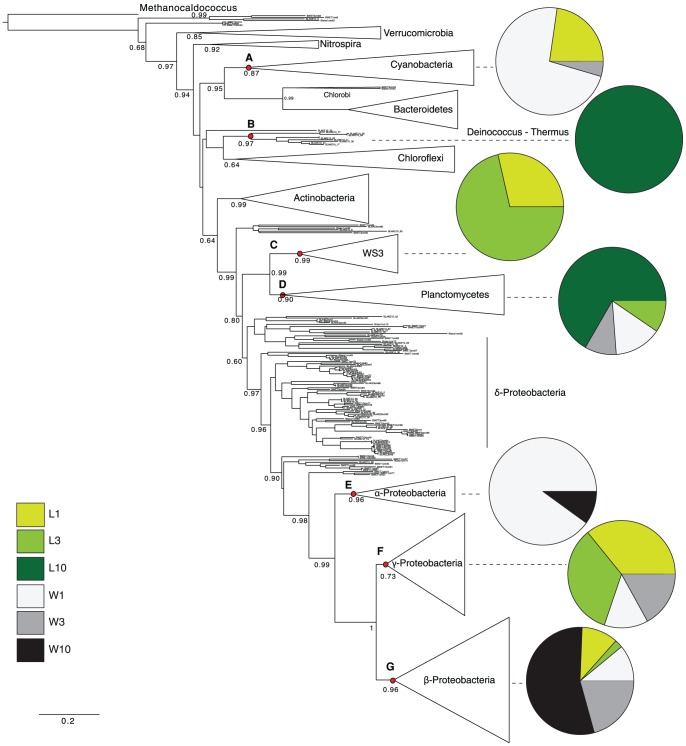
Maximum likelihood tree of bacterial 16S rRNA sequences. Major phyla, represented by triangles whose area is proportional to the number of sequences, were tested for lineage-specific differences using UniFrac. The lineage-specific analysis tests for each lineage whether the sequences have a different distribution among environments than does the tree overall and therefore highlight which lineage contributed to the differences observed (see Figure 3). Nodes A-D are significantly unevenly distributed between environments; *p*-values are: *p*_A_ = 1.1×10^−5^, *p*_B_ = 2.8×10^−3^, *p*_C_ = 5.8×10^−4^, *p*_D_ = 1.3×10^−3^, *p*_E_ = 1.0×10^−2^, *p*_F_ = 1.1×10^−19^. Pie charts connected to these nodes represent the distribution of environments within each lineage. Numbers adjacent to each node represent aLRT statistics (SH-like supports); only support values >0.50 are shown. Scale bar for branch lengths (expected number of substitutions per site) is shown.

To gain further insight into the origin of the vertical structure of the wetland and lake as well as into the differences among bacterial communities, we performed a lineage-specific analysis. Within the bacterial phylogeny, five phyla were found to contribute significantly to the differential structuring of the community: Cyanobacteria, Deinococcus-Thermus, the WS3 division, the Planctomycetes and the Proteobacteria (Figure 4). Cyanobacteria dominated the uppermost layers (1 and 3 cm), particularly in the wetland. Deinococci were only found in the lake at 10 cm; Planctomycetes also contributed to the overall difference between environments in the deepest lake sample. WS3 significantly contributed to the community structure of the lake sediments at 1 and 3 cm. Within the Proteobacteria, Alphaproteobacteria (especially *Sphingomonas*) and Betaproteobacteria (especially *Sulfuricella* and *Thiobacillus*) contributed to the wetland community structure, while Gammaproteobacteria (especially *Methylobacter*) contributed to the lake community particularly at 1 and 3 cm. Deltaproteobacteria were equally distributed among the environments sampled.

#### Archaea

Wetland and lake archaeal community structures were significantly different from one another (*p*≤0.01, *J* = 1.0) and also structured as a function of depth (Figure 3B). The split between 1–3 cm and 10 cm in the lake was only partially supported (*J* = 0.78), suggesting that depth may play a more critical role in structuring the Archaeal community in the first 3 cm (L1 is distinct from L3, *J* = 0.99). In the wetland, the community structures were less distinct in the first few cm (*J* = 0.55) than deeper in the sediment (*J* = 0.96). This clustering of the community in the wetland reflected the richness distribution previously observed with the rarefaction curves (Figure 2).

**Figure 5:**
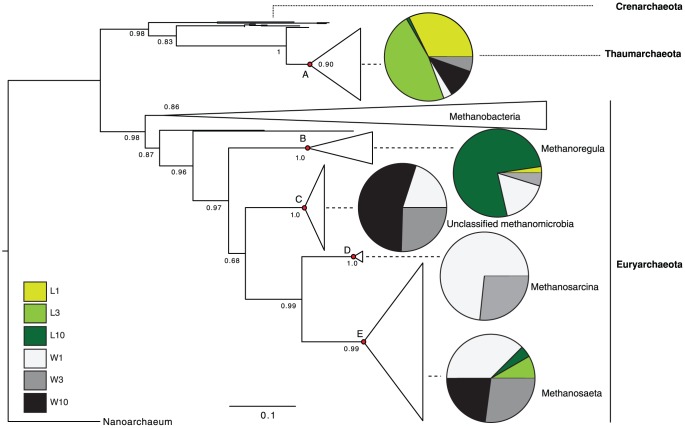
Maximum likelihood tree of archaeal 16S rRNA sequences. Major groups, represented by triangles whose area is proportional to the number of sequences, were tested for lineage-specific differences using UniFrac. The lineage-specific analysis tests for each lineage whether the sequences have a different distribution among environments than does the tree overall and therefore highlight which lineage contributed to the differences observed (see Figure 3). Nodes A-E are significantly unevenly distributed between environments; *p*-values are: *p*_A_ = 7.8×10^−19^, *p*_B_ = 9.3×10^−15^, *p*_C_ = 7.04×10^−43^, *p*_D_ = 7.6×10^−4^ and *p*_E_ = 7.4×10^−5^. Pie charts connected to these nodes represent the distribution of environments within that group of Archaea. Thicker lines correspond to member of the euryarchaeota. Numbers adjacent to each node represent aLRT statistics (SH-like supports); only support values >0.50 are shown. Scale bar for branch lengths is shown.

The lineage-specific analysis showed that differences between environments were attributed to the Euryarchaeota and the Crenarchaeota phyla, as well as to clones that were unclassified in both NCBI and the SILVA databases (Figure 5). The Crenarchaeota and the unclassified Archaea appeared mostly in lake surface sediments (1 and 3 cm); the SILVA database matched several clones to the tentative new archaeal phylum of the Thaumarchaeota, some of which were previously encountered in sediments of Lake Pavin, France [60]. Conversely, Euryarchaeota mostly appeared in the wetland and the vast majority of the clones were closely related to methanogens (Figure S3 in File S1), suggesting that the potential for methanogenesis is greater in the wetland, consistent with the RT-qPCR and diversity data. Within the Euryarchaeota phylum, most differences between environments were attributed to the Methanomicrobia class. *Methanoregula* significantly contributed to the lake community at 10 cm. *Methanosarcina* significantly contributed to the wetland environment at 1 and 3 cm. *Methanosaeta* contributed to the wetland community within the first 3 cm, with a dominance of clones identified as *Methanosaeta concilli*. A group of unclassified Archaea related to Methanomicrobia contributed significantly to differences between lake and wetland deep sediment community structures (Figure 5).

### *McrA* transcript sequence analyses

**Figure 6:**
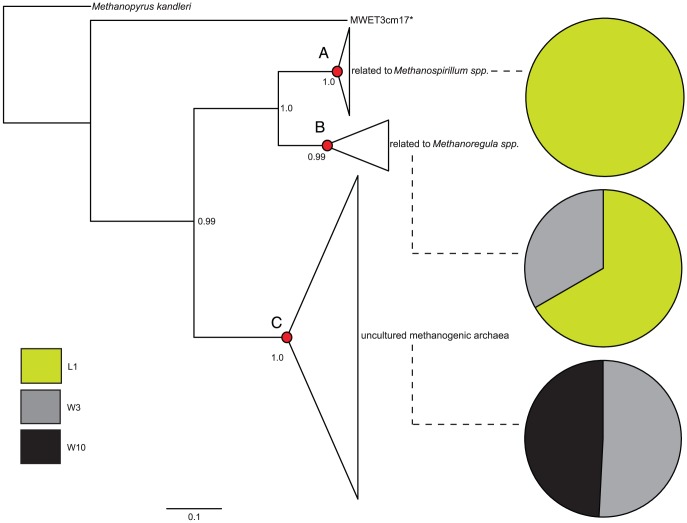
Maximum likelihood tree of *mcrA* sequences. Major groups, represented by triangles whose area is proportional to the number of sequences, were tested for lineage-specific differences using UniFrac. The lineage-specific analysis tests for each lineage whether the sequences have a different distribution among environments than does the tree overall and therefore highlight which lineage contributed to the differences observed (see Figure 3). Nodes A–C are significantly unevenly distributed between environments: *p*_A_ = 9.86×10^−13^, *p*_B_ = 3.50×10^−2^ and *p*_C_ = 1.97×10^−19^. Pie charts connected to these nodes represent the distribution of environments within that group of Archaea. Numbers adjacent to each node represent aLRT statistics; only support values >0.90 are shown. Scale bar for branch lengths is shown. *: *Methanobacterium* sp.

*McrA* gene transcripts cluster analysis revealed that both depth and environment contributed to structuring the community, with each habitat separating away from the others 100% of the time (*J* = 1.0, Figure 3C). The lineage-specific analysis enabled us to identify members of the active methanogenic community. Node A (Figure 6; *p*_A_ = 9.86×10^−13^) was comprised almost entirely of lake-derived sequences and represented species related to *Methanospirillum* spp. Node B contained sequences related to *Methanoregula* spp. and was comprised largely of lake surface sequences (*p*_B_ = 3.50×10^−2^). Finally, a group of uncultured methanogenic Archaea, distantly related to *Methanocella* spp. (node C; *p*_C_ = 1.97×10^−19^), was uniquely found in wetland sediment. Most of the *mcrA* diversity found in the wetland did not match any known sequences in the databases.

As *mcrA* is a functional gene, the protein structure could be estimated to test for differences in the three-dimensional (3D) structure and corresponding presence/absence of protein motifs as a function of environment. We observed a 7-amino acid indel between lake and wetland protein alignments (Figure S4 in File S1). Most of the amino acid sequences from the lake (73%) contained a very conserved motif “PKDKVKP” corresponding to a loop on the surface of the protein similar to *Methanospirillum* spp in our 3D model (Figure S5 in File S1). All wetland sequences lacked this loop. Selective pressures unique to the lake may have led to the evolution (emergence or retention) of this loop. As all database searches using the highly-sensitive HHblits failed to identify any match containing this motif, it may represent a synapomorphy singular to this lake environment whose function is yet to be characterized.

## Discussion

By extracting and amplifying environmental RNA from sediment cores collected in a high Arctic polar desert, we demonstrate the possibility of characterizing bacterial and archaeal community structures from two types of freshwater sediments, a lake and a wetland, as a function of depth. Our approach also permits the relative quantification of microbial activity by employing transcript abundance as a proxy; other studies have shown the existence of a relationship between transcript abundance and microbial activity (*e.g.*, Freitag et al., 2010), hereby laying ground for the approach adopted here. Our results show that these freshwater sediment communities are vertically structured, and that although richness and diversity indices were similar between the lake and wetland when examining only 16S rRNA transcripts, they differed when *mcrA* transcripts (a proxy for active methane cycling) were also considered.

### Arctic freshwater sediments support highly diverse and contrasted microbial communities

Although Cornwallis Island is a polar desert, diversity and richness for both Archaea and Bacteria were greater than previously found in the Norwegian high Arctic permafrost [6], in the Lena delta, Siberia [61] and even in more temperate regions such as Lake Pavin, France [60], [62] or the Florida Everglades [63] when using similar clone library approaches based on single-core samples. Likewise, methanogen richness and diversity estimates (based on *mcrA* transcripts) were comparable to those observed for methanogens (based on 16S rRNA) in temperate sites across the central to northern Appalachian Mountain region [64] or the Everglades [63].

In addition to this high microbial diversity and richness, critical differences exist between our two freshwater environments in terms of (i) species diversity (Table 1 and Figure 2) and (ii) community structure (Figures 3–6). These differences may reflect the extensive diversity of metabolic strategies required to exploit or to contribute to the various geochemical gradients encountered with depth in these two environments. Although we did not perform incubation experiments to quantify methane production and destruction or determine the methanogenic pathways involved, our results allow us to predict which environmental variables that may have contributed to shape these differences.

First, the number of active microbes in lake sediments, as inferred by *glnA* and *mcrA* transcript abundance, decreased with depth, which may result from carbon limitation, availability of terminal electron acceptors and/or changes in lake sediment geochemistry. Indeed, Char Lake is deep, highly oligotrophic [65] and only mixes briefly during the summer [21]. Char Lake sediment has very low carbon, nitrogen and phosphorus levels, which are at a maximum at the surface and decline sharply with depth [24], [66]. Moreover, labile algal-derived carbon increases several folds in concentration in the top layer of lake sediment [67]. This increase in labile carbon at the surface of lake sediments corroborates well with Char Lake recently experiencing a reduced summer ice cover leading to increased primary productivity [22]. This greater content of labile carbon in recently deposited sediments is consistent with our RT-qPCR results, which suggest a greater microbial activity at the surface.

On the other hand, Small Lake Wetland is expected to contain much more organic carbon. Two lines of evidence support this expectation. First, a survey of wetlands in the same area revealed that carbon content range between 7–70% [13], as compared to 1–2% in Char Lake surface sediment [24], [66]. Second, organic matter in Char Lake sediment originates predominately from algal sources [67], while carbon in wetland sediment is more a mix of terrestrial and algal sources [23], [26] (see photographs S1 and S2 in File S1). Consistent with this expectation of higher organic carbon in the wetland, we show here that the abundance of active microbes increased with depth in wetland sediments (Figure 1), suggesting an appropriate supply of carbon to support microbial activity across sediment depth. Surface sediments are likely oxygenated (*e.g*., due to the presence of cyanobacterial mats and a shallow depth of overlying water), impeding anaerobic processes such as methanogenesis and anaerobic methane oxidation, explaining why *mcrA* transcript abundance at the wetland surface reached our limit of detection.

Last, as for Bacteria, archaeal community structures were different between lake and wetland environments, but they appeared to be less dependent on depth, suggesting that vertical redox gradients cannot solely account for differences in species distribution. We further posit that archaeal community structures differed between lake and wetland environments most likely because of the nature of the carbon substrates available, rather than the nature of the terminal electron acceptors.

### Reconciling archaeal 16S rRNA and *mcrA* data

Our transcriptomics results revealed that the abundance of active microbes over depth differed between lake and wetland environments. These differences paralleled those observed from *mcrA* and 16S rRNA sequence analyses and likely originated from the fundamental difference in the current and past limnological properties of Char Lake and Small Lake Wetland.

While *mcrA* transcripts were only found within lake sediments at a depth of 1 cm and deeper within wetland sediments (3 and 10 cm), 16S rRNA data indicated presence of methanogenic Archaea at all depths, both in wetland and lake sediments. Consequently, it can be inferred that active methane cycling is highly localized in each environment, being limited to certain depths despite the presence of methanogens at all depths. This difference between active (inferred by *mcrA* transcript analyses) and potential methane cycling (with methanogenic players identified by 16S rRNA) may be explained in terms of the geochemical dynamics specific to each environment.

The wetland is a transient system, drying up some summers, leading to surface sediments that are occasionally aerated. This is consistent with our inability to detect active methanogenesis in wetland surface sediment. In Char Lake, acid volatile sulfide profiles in sediments, used to infer S redox cycling [24], suggest that oxygen can penetrate, at least seasonally, into surficial sediments. However, because of enhanced algal production in the water column, sediments collected during the summer would exhibit a large oxygen demand. This expectation is consistent with our detection of *mcrA* transcripts at the surface, suggesting anoxic conditions and active methane cycling at the sediment surface. Furthermore, previous studies have shown that methanogenic precursors vary considerably, both spatially and temporally [68] and that sediments are spatially chemically heterogeneous: the presence of micro-oxic niches in otherwise anoxic sediments can be expected, for instance due the activity of burrowing macrofauna [9], [69]. Consequently, the discrepancy we observed between *mcrA* and 16S microbial community structures may thus reflect active methane cycling at the time of sampling (*mcrA* data) and a combination of both active members from the current season as well as dormant members from previous seasons when conditions differed (16S rRNA data).

Finally, based on transcript sequence data, our results support a greater diversity, and hence significance, of methane cycling in the wetland as compared to the lake. This parallel is consistent with the positive correlation that exists between the diversity of methanogenic communities and rates of methane production [64]. Therefore, in polar deserts, a wetland may have a higher potential for methane cycling than a lake, not just because of a higher abundance of *mcrA* transcripts, but also because of a greater genetic diversity, which potentially reflects a greater diversity of methane production and oxidation pathways. Given that both lakes and wetlands have the potential to be sites for active methane cycling, additional work is required to estimate net methane emissions from these polar desert aquatic systems.

Our survey of the molecular diversity of microbes thriving in polar desert lake and wetland sediments highlights a very complex community structure with as yet uncharacterized microbial players inferred to possess diverse methane cycling strategies. Furthermore, some of the methanogenic archaea suspected to be active both in lake and wetland sediments have recently been tagged as potential mercury methylators [17], opening up a new field of investigation into the interplay between methane and mercury cycles in these already fragile environments. Our molecular data also support the model that wetlands set in polar deserts may be sites conducive to methane cycling, even if the fate of the methane produced remains to be investigated. Finally, we demonstrate the importance of combining targeted functional gene transcript analyses with environmental genomics in order to gain a functional perspective on the composition of microbial communities, particularly when studying transient environments in the high Arctic.

## Supporting Information

File S1 **Tables S1–S3, Photographs S1 and S2, Figures S1–S5.** Table S1A. Basic water chemistry for the wetland. Table S1B. Basic water chemistry for the lake. Table S2. Forward and Reverse PCR primers used for each target sequence. Table S3. p-values for SH-like comparison. Photograph S1. Picture of Char Lake. Photograph S2. Picture of the wetland. Figure S1. Summary of the experimental procedure, including RNA treatments and quality controls. Figure S2. Cumulative bacterial clonal frequency of phyla identified using 16S rRNA clone libraries. Bottom panel details the most abundant phylum. Figure S3. Cumulative archaeal clonal frequency of phyla identified using 16S rRNA clone libraries. Bottom panel details the most abundant class. Figure S4. Amino acid alignment of selected lake and wetland sequences showing the PKDKVKP conserved motif for the lake sequences. Figure S5. Left and bottom view of the McrA protein in a model lake sequence (A, B), *Methanospirillum hungatei* (C,D) and a model wetland sequence (E,F). Region highlighted in blue represents the insert present in lake sequences that is modified and much shortened in wetland sequences. A comparable insert exists in the *Methanospirillum hungatei* McrA protein.
(PDF)
