# Supplementary material for: Microbial Community Structure in Lake and Wetland Sediments from a High Arctic Polar Desert Revealed by Targeted Transcriptomics
Source: PLoS One. 2014 Mar 3;9(3):e89531. doi: 10.1371/journal.pone.0089531 (PMC3940601; doi:10.1371/journal.pone.0089531)

Microbial community structure in lake and wetland sediments from a high Arctic polar desert revealed by targeted transcriptomics

Supporting online information

Magdalena K Stoeva[1], Stéphane Aris-Brosou[1,2], John Chételat[3], Holger Hintelmann[4], Philip Pelletier[1] and Alexandre J. Poulain[1*].

[1] Department of Biology, University of Ottawa, Ottawa ON, Canada
[2] Department of Mathematics and Statistics, University of Ottawa, Ottawa ON, Canada
[3] Environment Canada, National Wildlife Research Centre, Ottawa, ON, Canada
[4] Department of Chemistry, Trent University, Peterborough, ON, Canada

[*]Correspondence: A.J. Poulain, Department of Biology, University of Ottawa, 30 Marie Curie, Ottawa ON, K1N 6N5, Canada. Phone: 613-562-5800 x2373; Fax: 613-562-5486; e-mail: apoulain@uottawa.ca

Supporting online information:

Supporting Tables: 3
Supporting Photographs: 2
Supporting Figures: 5

Supplementary Tables

Table S1A. Basic water chemistry for the wetland.

	pH	Conductivity (μS.cm^{-1})	Temperature (^{0}C)	Ref.
Wetland	8.13	423	12.2	This study

Table S1B. Basic water chemistry for Char Lake

	pH	Conductivity (μS.cm^{-1})	Temperature (^{0}C)	Ref.
Char lake surface	7.92	264	5.6	This study
Char lake 14 m	8.19	263	5.3	This study

Table S2. Forward and Reverse PCR primers used for each target sequence

**Sequence**	**Forward Primer**		**Reverse Primer**	
Bacteria 16S	27F	5'-AGA GTT TGA TCM TGG CTC AG-3'	907R	5'-CCG TCA ATT CMT TTR AGT TT-3'
Archaea 16S	109F	5'-ACK GCT CAG TAA CAC GT-3'	915R	5'-GTG CTC CCC CGC CAA TTC CT-3'
mcrA	ME1	5'-GCM ATG CAR ATH GGW ATG TC-3'	ME2	5'-TCA TKG CRT AGT TDG GRT AGT-3'
glnA	glnA-F	5'-GAT GCC GCC GAT GTA GTA-3'	glnA-R	5'-AAG ACC GCG ACC TTY ATG CC-3'

Table S3. p-values for SH-like comparison

Models	Trees	Archaea	Bacteria	
		GTR + Γ*	GTR + Γ+I*	GTR + Γ
FastTree vs. PhyML	τ_1**	-	-	-
	τ_2	p=0.4884	p=0.4991	p=0.4985
FastTree 'env.' vs. 'env. + known'	τ_1**	-	-	-
	τ_2	p=0.4929	p=0.4889	p=0.4853

* Model selected by AIC in jModelTest
** Maximum likelihood tree
τ_1: tree estimated with FastTree on environmental sequences; τ_2: tree estimated with PhyML (top rows) or FastTree with known sequences (bottom rows).

Supplementary Field Site Pictures.

Photograph S1. Picture of Char Lake; east view.

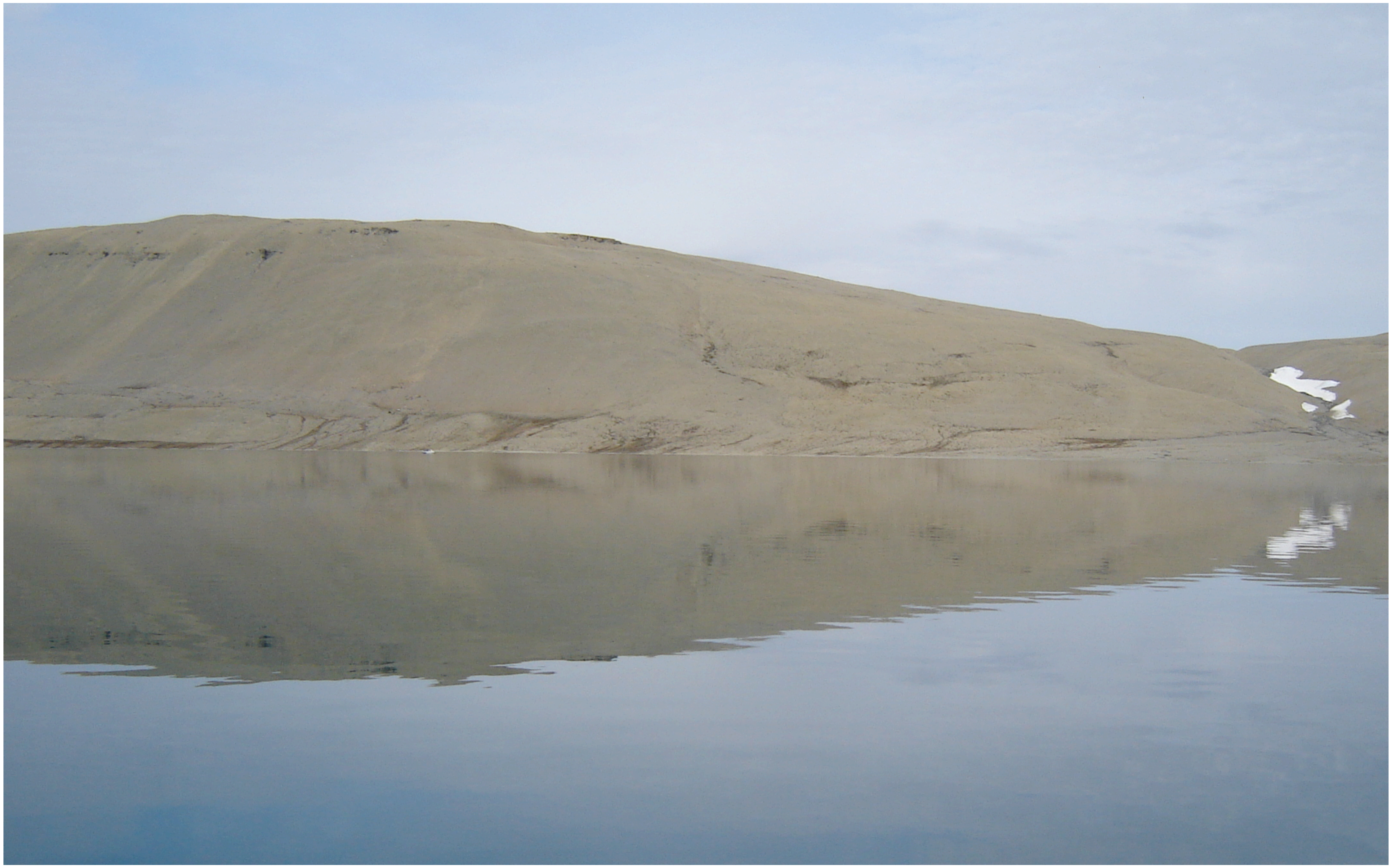

Photograph S2. Picture of the wetland.

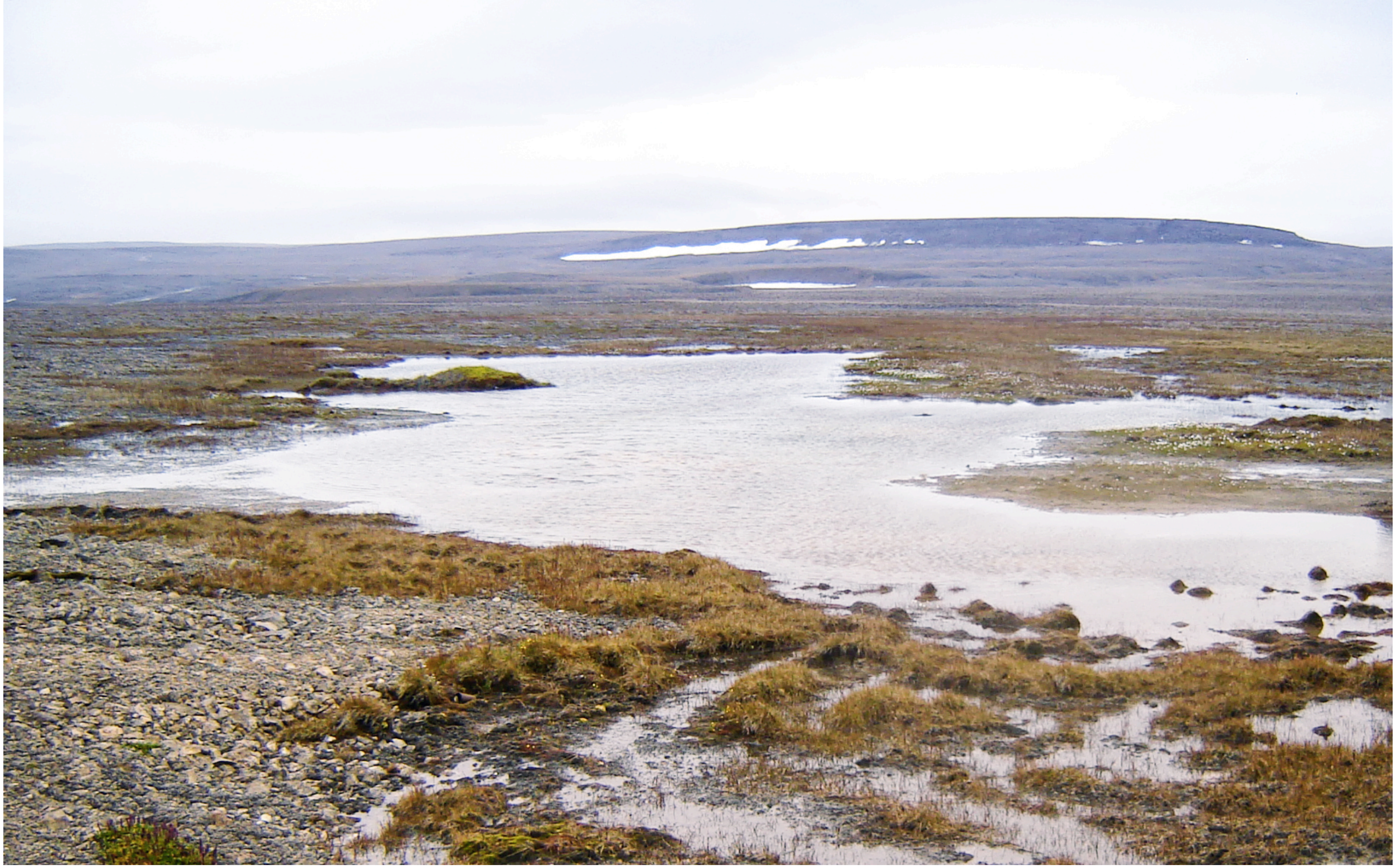

Supplementary Figures

Figure S1. Summary of the experimental procedure, including RNA treatments and quality controls.

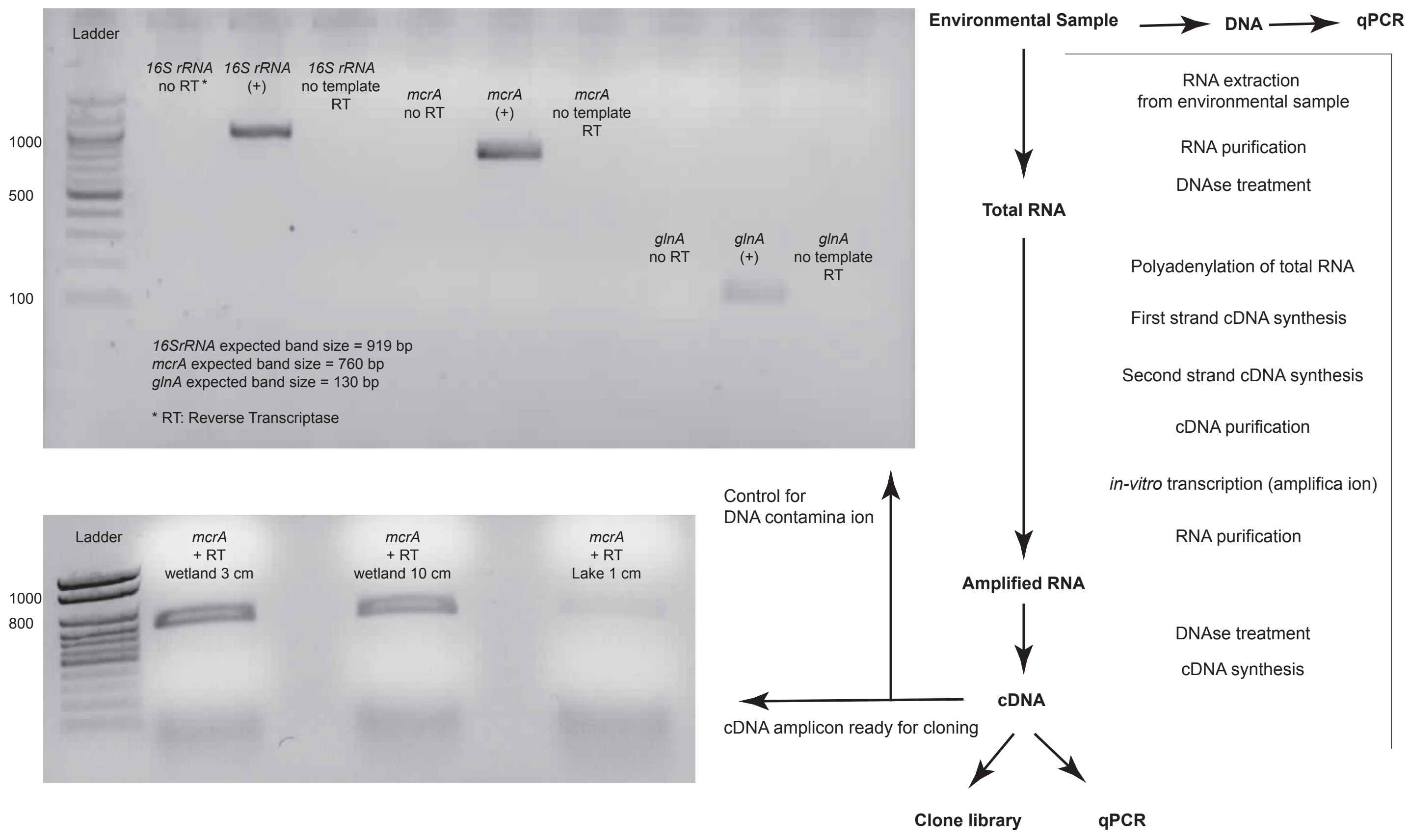

Figure S2. Cumulative bacterial clonal frequency of phyla identified using 16S rRNA clone libraries. Bottom panel details the most abundant phylum.

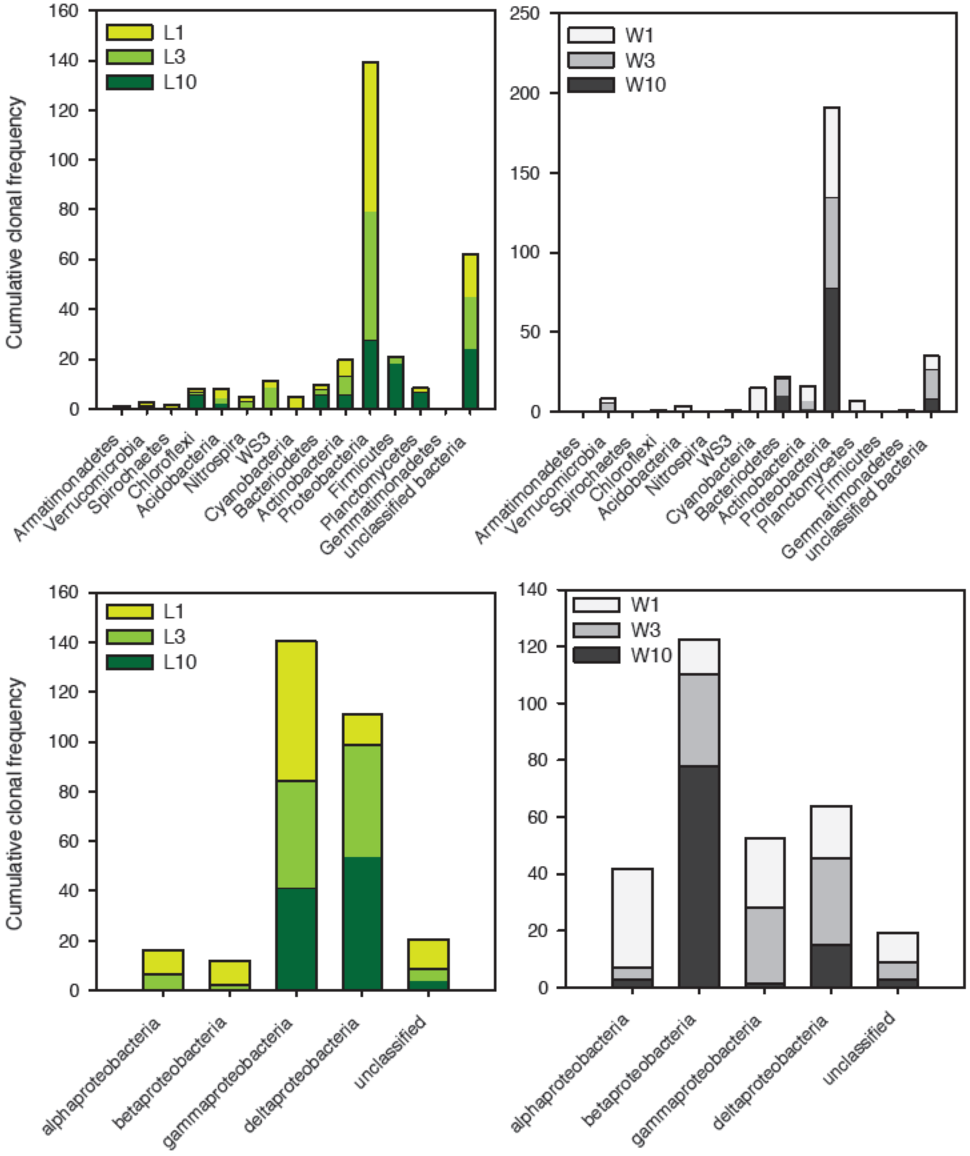

Figure S3. Cumulative archaeal clonal frequency of phyla identified using 16S rRNA clone libraries. Bottom panel details the most abundant class.

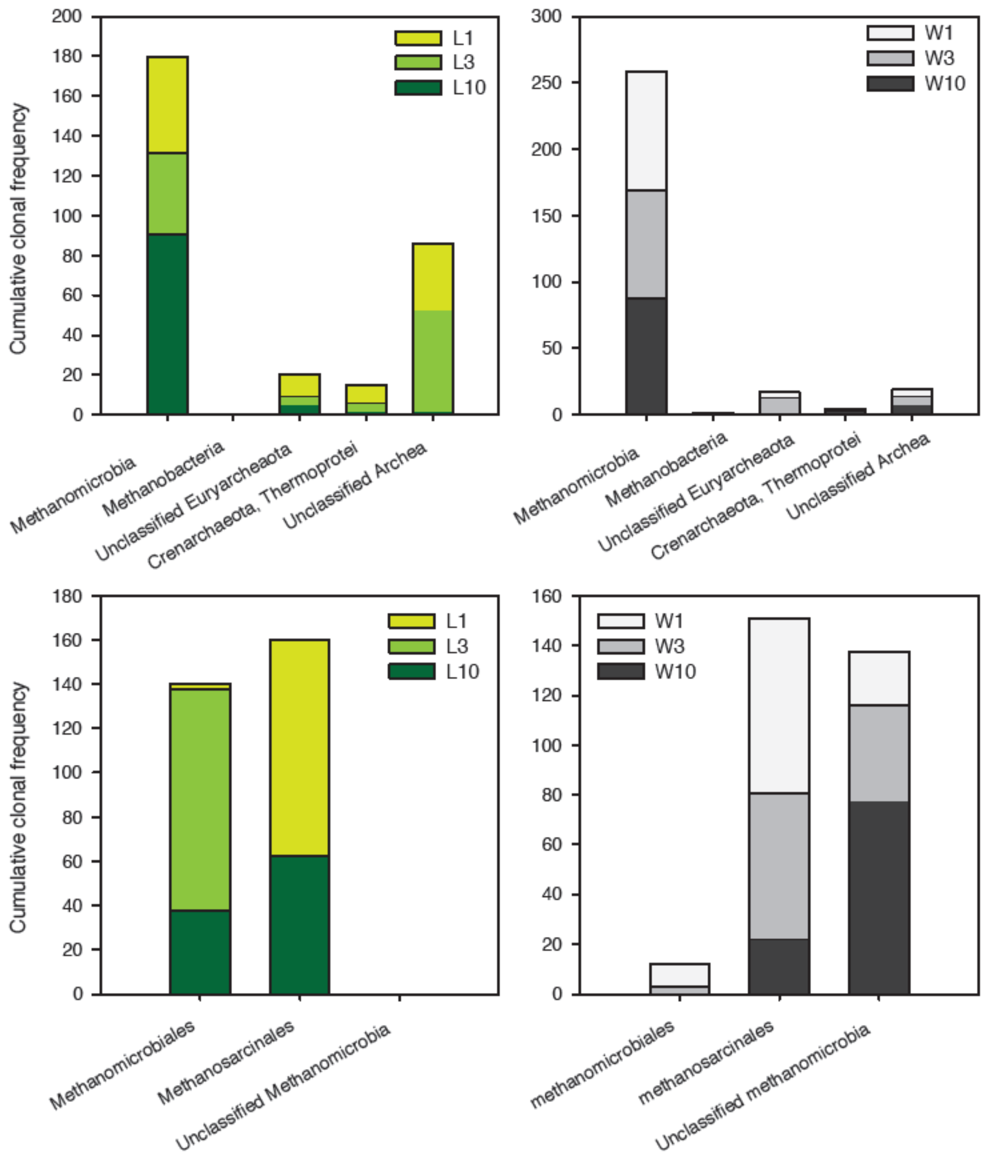

Figure S4. Amino acid alignment of selected lake and wetland sequences showing the PKDKVKP conserved motif for the lake sequences.

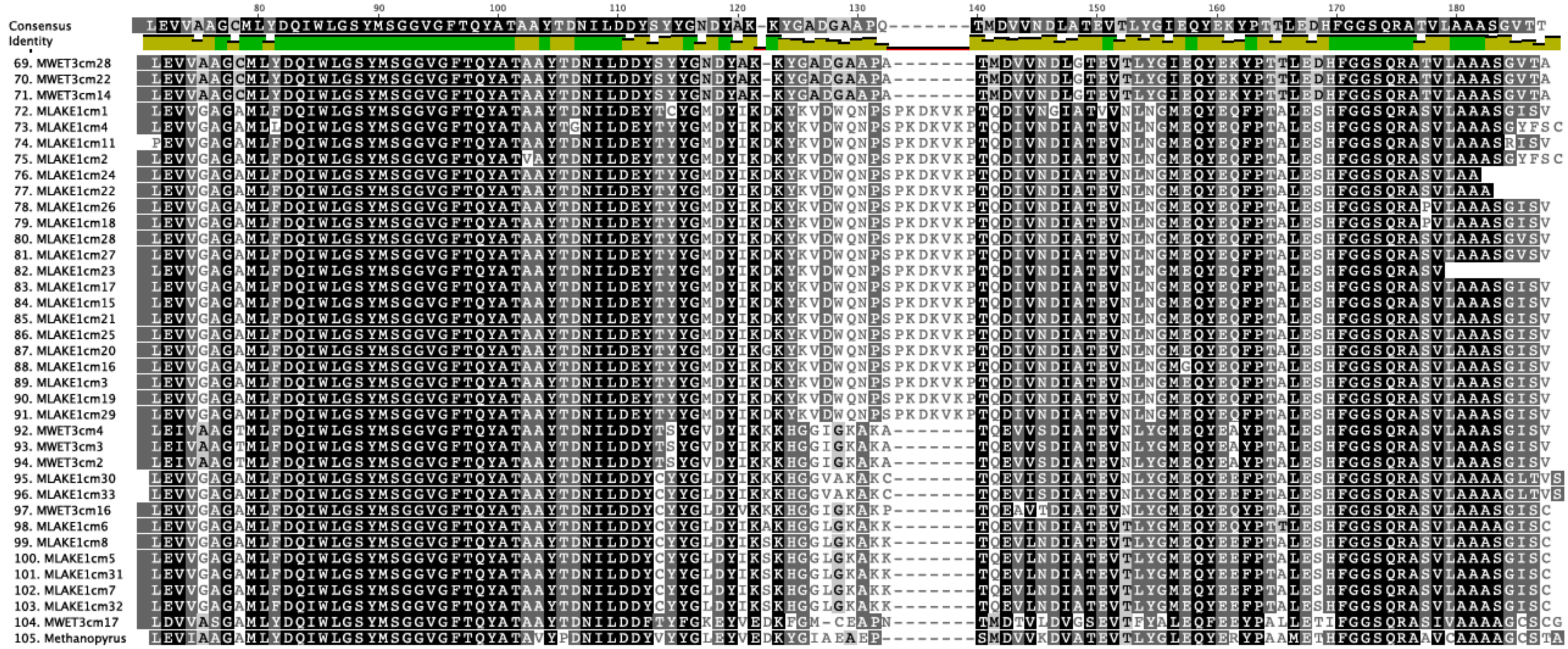

Figure S5. Left and bottom view of the McrA protein in a model lake sequence (A, B), *Methanospirillum hungatei* (C,D) and a model wetland sequence (E,F). Region highlighted in blue represents the insert present in lake sequences that is modified and much shortened in wetland sequences. A comparable insert exists in the *Methanospirillum hungatei* McrA protein.

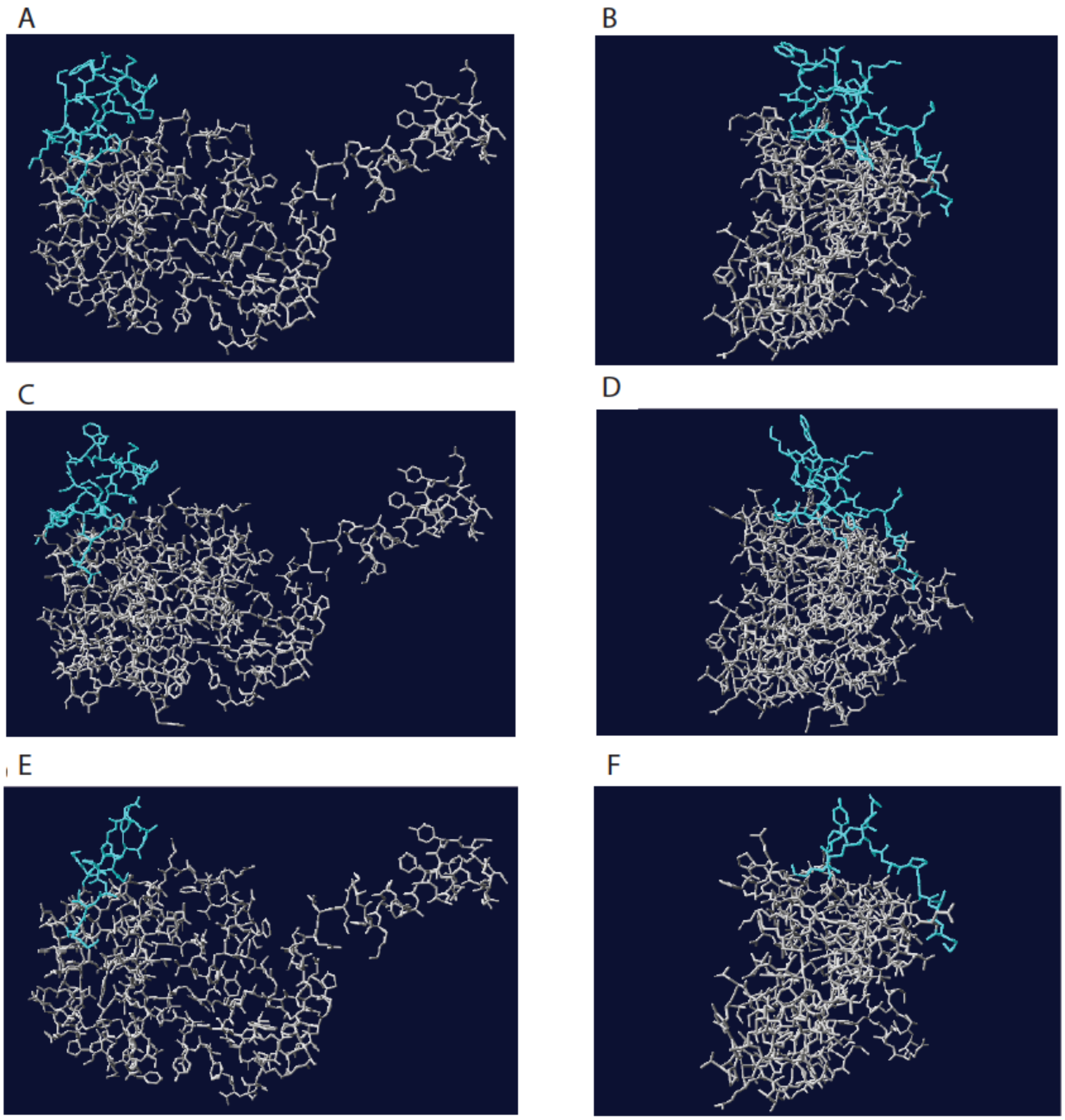

Supplement: File S1 — Tables S1–S3, Photographs S1 and S2, Figures S1–S5. Table S1A. Basic water chemistry for the wetland. Table S1B. Basic water chemistry for the lake. Table S2. Forward and Reverse PCR primers used for each target sequence. Table S3. p-values for SH-like comparison. Photograph S1. Picture of Char Lake. Photograph S2. Picture of the wetland. Figure S1. Summary of the experimental procedure, including RNA treatments and quality controls. Figure S2. Cumulative bacterial clonal frequency of phyla identified using 16S rRNA clone libraries. Bottom panel details the most abundant phylum. Figure S3. Cumulative archaeal clonal frequency of phyla identified using 16S rRNA clone libraries. Bottom panel details the most abundant class. Figure S4. Amino acid alignment of selected lake and wetland sequences showing the PKDKVKP conserved motif for the lake sequences. Figure S5. Left and bottom view of the McrA protein in a model lake sequence (A, B), Methanospirillum hungatei (C,D) and a model wetland sequence (E,F). Region highlighted in blue represents the insert present in lake sequences that is modified and much shortened in wetland sequences. A comparable insert exists in the Methanospirillum hungatei McrA protein. (PDF) [file pone.0089531.s001.pdf]
